# A Systematic Review of Delta-9-Tetrahydrocannabinol (∆9-THC) in Astrocytic Markers

**DOI:** 10.3390/cells13191628

**Published:** 2024-09-29

**Authors:** Christian Ramos-Jiménez, Sarah Petkau, Romina Mizrahi

**Affiliations:** 1 Integrated Program in Neuroscience, McGill University, Montreal, QC H3A 1A1, Canada; christian.ramosjimenez@mail.mcgill.ca; 2 Clinical and Translational Sciences Lab, Douglas Research Centre, Montreal, QC H4H 1R3, Canada; sarah.petkau.comtl@ssss.gouv.qc.ca; 3 Department of Psychiatry, McGill University, Montreal, QC H3A 1A1, Canada

**Keywords:** ∆9-THC, cannabis, marihuana, astrocytes, GFAP, GLAST, nestin, neuroinflammation, astrogliosis

## Abstract

Background: Astrocytic reactivity in substance use disorders (SUDs) has been extensively studied, yet the molecular effect of delta-9-tetrahydrocannabinol (∆9-THC, the main psychoactive compound in cannabis) on glial cells, especially astrocytes, remains poorly understood. Exploring ∆9-THC’s impact on astrocytic markers can provide insight into its effects on brain functions such as homeostasis, synaptic transmission, and response to neuronal injury. This systematic review synthesizes findings from studies investigating ∆9-THC’s impact on astrocytic markers. Methods: A systematic review was conducted using EMBASE, Medline, and PsychoInfo via the OvidSP platform. Studies reporting astrocytic markers following ∆9-THC exposure in animals and humans were included. Data were extracted from twelve eligible full-text articles, and the risk of bias was assessed using the Systematic Review Center for Laboratory Animal Experimentation. Results: This research identified several astrocytic markers, including glial fibrillary acidic protein (GFAP), nestin, and glutamate–aspartate transporter (GLAST). Both GFAP and nestin expressions increased in adulthood following adolescence and adult ∆9-THC exposure. An increase in GLAST expression was also noted during early development after ∆9-THC exposure. Conclusions: This review indicates varying levels of astrocytic reactivity to ∆9-THC across different developmental stages, including adolescence and adulthood. ∆9-THC appears to impact maturation, particularly during early developmental stages, and exhibits sex-dependent effects.

## 1. Introduction

Cannabis is widely used globally [1]. Late adolescence, with a median onset age of 18–19 years, marks the period of the highest prevalence of recreational cannabis use [2,3]. Evidence indicates that regular and heavy cannabis use increases the risk of developing various mental disorders and health problems, including cognitive impairment [4], mood and anxiety disorders [5,6], psychosis [7], suicide [8], and substance use disorders [2,9].

Cannabis, also known as marijuana or weed, belongs to the Cannabaceae family and contains over 500 distinct chemical compounds, including more than 100 cannabinoids. The most well-known cannabinoids are cannabidiol (CBD) and delta-9-tetrahydrocannabinol (∆9-THC) [10]. CBD exhibits potential therapeutic benefits, including analgesic, anti-epileptic, and anti-inflammatory effects, whereas Δ9-THC, the psychoactive constituent responsible for the euphoric effects of cannabis “high”, has demonstrated efficacy in treating chemotherapy-induced nausea [11]. It activates the endocannabinoid system via the brain’s endocannabinoid type 1 receptor (CB_1_R), with effects varying based on dose, usage, and exposure duration [12]. CB_1_R, a G-protein coupled receptor, is abundantly found in the brain, primarily residing on presynaptic terminals of GABAergic and glutamatergic neurons, with some presence in astrocytes [13]. CB_1_Rs regulate neural excitability by modulating the release of gamma-aminobutyric acid (GABA) and glutamate through a retrograde signaling mechanism [14]. Despite their lower abundance compared to GABAergic and glutamatergic neurons, CB1Rs in astrocytes are believed to significantly influence working memory processes [15].

Astrocytes, the predominant subtype of glial cells in the brain, perform essential functions in neuroplasticity, neurodevelopment, maturation, central nervous system homeostasis, and the regulation of neural circuits [16,17]. Astrocytes are activated in response to oxidative stress and neuroinflammation, processes that can disrupt normal cellular function [18,19]. Prolonged or uncontrolled astrocyte activation results in irreversible alterations in their morphology, gene expression, and function, thereby contributing to adverse effects across different regions [18,20]. Some preclinical studies suggest that repeated ∆9-THC exposure during adolescence induces these morphological changes in astrocytes and subsequently increases the release of astrocyte markers such as the glial fibrillary acidic protein (GFAP), commonly used as a marker of astrocyte activation, and nestin [21,22]. However, the current literature lacks consensus on changes in GFAP levels; some studies suggest that ∆9-THC administration decreases GFAP levels, presenting an opposite view [23,24,25,26,27]. These contrasting effects on GFAP expression highlight the complex interplay between ∆9-THC and astrocyte function, underscoring the need for further research into the effects of ∆9-THC on astrocyte biology. To address this gap, we conducted a systematic review of animal and human studies which examined the effects of ∆9-THC on astrocytic markers.

## 2. Materials and Methods

**Figure 1:**
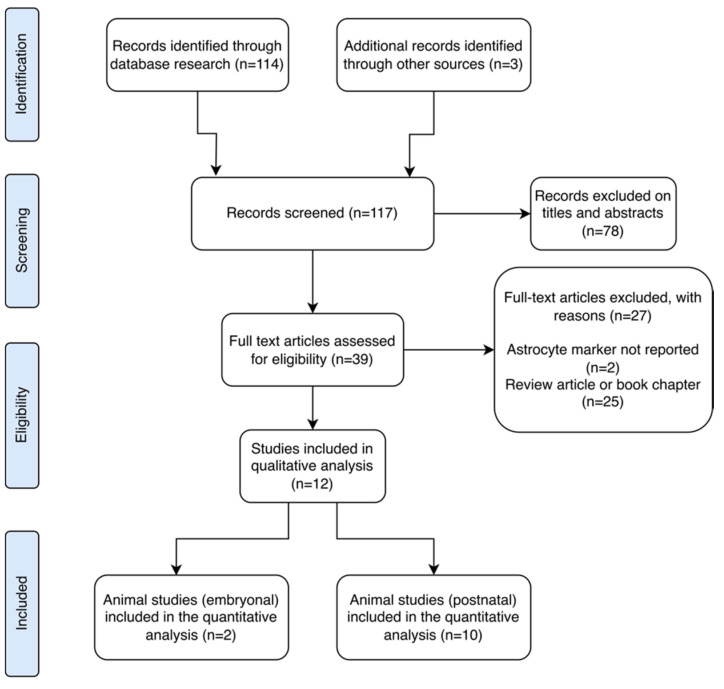
PRISMA flow diagram showing data extraction process.

Aligned with the Preferred Reporting Items for Systematic Reviews and Meta-Analyses (PRISMA) guidelines, our main aim was to identify scientific literature examining the effects of ∆9-THC on astrocytes in both animal and human models. PROSPERO registration was not included since data extraction was performed after the registration. We reviewed all studies reporting outcomes related to astrocyte activity assessed through any astrocyte marker following exposure to ∆9-THC (Figure 1).

### 2.1. Eligibility Criteria

The following inclusion criteria were applied to select studies: (1) retrospective and prospective papers published in English, (2) studies conducted on humans or animals from 1946 to 2024, (3) investigations into the acute or long-term effects of ∆9-THC administration, and (4) studies assessing a broad spectrum of molecular markers associated with astrocyte reactivity. These markers include cytoskeleton markers (e.g., glial fibrillary acidic protein (GFAP), nestin, synemin, and vimentin), metabolic markers (e.g., aldolase C (ALDOC), brain lipid binding protein (BLBP), and monoamine oxidase B (MAO-B)), transcription factors (e.g., nuclear factor of activated T cells (NFAT)), and channel transporters (e.g., excitatory amino acid transporter 1 and 2 (EAAT1, EAAT2), and the inwardly rectifying K+ channel (Kir) subtype Kir4.1). Exclusion criteria included the following: (1) papers not written in English, (2) studies exclusively focusing on other synthetic cannabinoids rather than ∆9-THC, and (3) studies where the outcome was not reported.

### 2.2. Literature Search

The systematic search was undertaken on the 18th of March 2024 using EMBASE, Medline, and PsychoInfo through the OvidSP platform. An extensive literature search was performed using the following terms: (“cannabis” or “marijuana” or “marihuana” or “tetrahydrocannabinol” or “∆9-THC” or “THC” or “∆9-tetrahydrocannabinol” or “dronabinol”) and (“astrocyte reactivity” or “astrocytosis” or “astrogliosis” or “reactive gliosis” or “astrocyte activation”) and (“glial fibrillary acidic protein” or “GFAP” or “nestin” or “synemin” or “vimentin” or “aldolase C” or “ALDOC” or “brain lipid binding protein” or “BLBP” or “monoamine oxidase B” or “MAO-B” or “nuclear factor of activated T cells” or “NFAT” or “excitatory amino acid transporter 1” or “EAAT1” or “excitatory amino acid transporter 2” or “EAAT2” or “inwardly rectifying K^+^ channel” or “Kir4.1”). The terms “cannabis”, “marihuana”, and “marijuana” were included in this search to capture all manuscripts that studied ∆9-THC.

### 2.3. Data Extraction

The records from the database were imported and saved to reference management software Zotero version number 6.0.30. All the studies identified were extracted into a spreadsheet for comprehensive review. The primary outcome of interest, focusing on astrocyte markers, was assessed across interventional/exposed cohorts and/or between exposed and non-exposed control groups. These outcomes were then compared and grouped into postnatal (in vitro and in vivo) and embryonal animal models.

### 2.4. Risk of Bias and Quality Assessment

**Figure 2:**
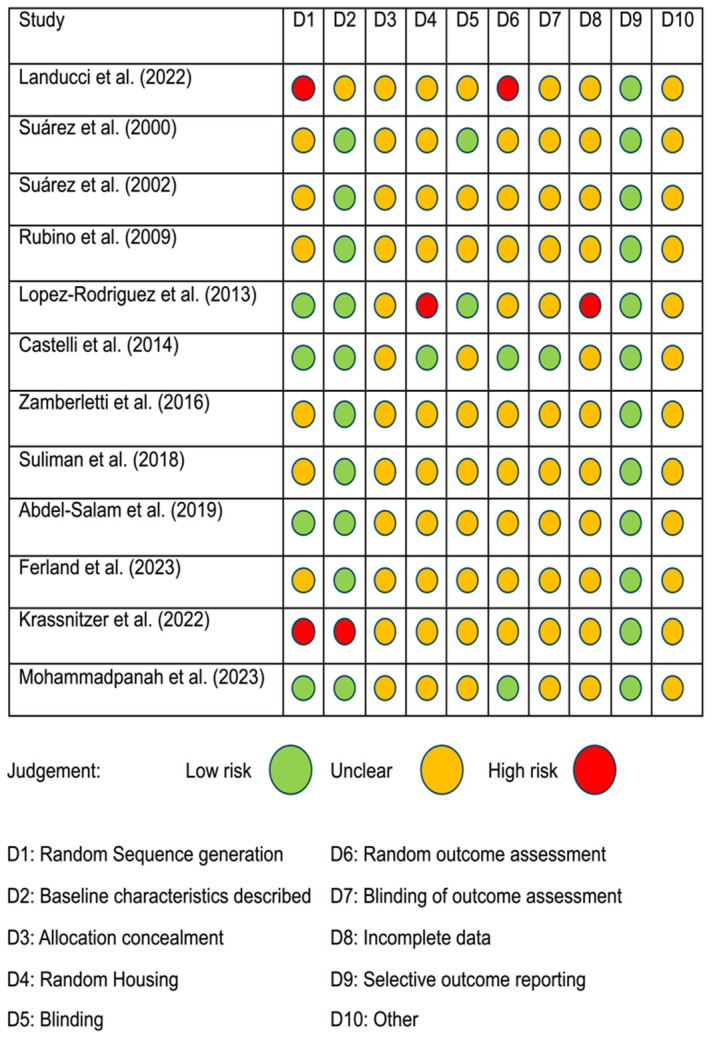
Assessment of risk of bias for each study (rows) and criterion (columns) using SYRCLE’s (Systematic Review Centre for Laboratory Animal Experimentation) risk of bias tool [21,22,23,24,25,26,27,29,30,31,32,33].

To ensure consistency and minimize discrepancies in assessing the risk of bias (RoB), we employed the Systematic Review Center for Laboratory Animal Experimentation (SYRCLE) tool. This tool consists of specific questions derived from the reviewed papers [28], with each paper categorized as having a low, high, or unclear risk of bias. Two authors (C.R.J. and S.P.) independently used this method to address the SYRCLE risk of bias questions. Any disagreements in their assessments were resolved through co-authors discussion until consensus was achieved. Further details regarding the classification and specific criteria used to evaluate RoB are provided in Figure 2.

## 3. Results

### 3.1. Study Selection

A total of 117 records were initially identified through database searches and other resources. The main findings from these records are summarized in Table 1. After screening by title and abstract, 78 records were excluded. Subsequently, 27 studies were further excluded for reasons such as lack of reporting on astrocyte markers, involving synthetic cannabinoid receptor agonists, or being review articles. Ultimately, 12 studies met the criteria for qualitative analysis. Among these, one study was conducted in vitro, two studies focused on animal research in embryological contexts, and one specifically addressed early development. Additionally, five studies involved adolescent subjects, with one reporting translational outcomes in humans, while three studies focused on adults. No discernible human outcomes related to astrocytic markers were identified in this review. These findings highlight the rigorous selection process undertaken to ensure the quality and relevance of the included studies for our analysis (Figure 1). 

The included studies primarily investigated various aspects of astrocyte reactivity in response to ∆9-THC/dronabinol exposure, focusing on factors such as astrocyte morphology and the expression of astrocytic markers across different brain regions in animal models. Notably, investigations into GFAP levels following ∆9-THC administration were conducted, which spanned both in vitro postnatal and in vivo embryonal studies. Additionally, studies examining other astrocytic markers such as glutamate–aspartate transporter (GLAST) and nestin provided insights into the effects of ∆9-THC exposure during early development and adolescence, respectively.

### 3.2. Astrocytic Marker Outcome Measures in Response to ∆9-THC Application/Administration in Animal Models

Twelve studies explored astrocytic markers in animal models (rats and mice), following ∆9-THC administration, using both embryonal/postnatal in vivo and in vitro methods. Various techniques such as immunohistochemistry, in situ hybridization (ISH), fluorescent in situ hybridization (FISH), enzyme-linked immunosorbent assay (ELISA), and Western blot were compared (Table 1). Notably, one study conducted a translational analysis, juxtaposing findings from an animal model with human data, and highlighted the significance of cross-species validation in understanding the effect of ∆9-THC exposure.

#### 3.2.1. Animal Study of GFAP Levels of In Vitro ∆9-THC Exposure

In a recent study by Landucci et al. [23], the prolonged effects of ∆9-THC and CBD on hippocampal slices from rats were investigated in vitro. The study revealed that after 72 h of ∆9-THC exposure, GFAP expression decreased in the CA1 stratum pyramidale (SP) and stratum radiatum (SR) of the hippocampus compared to controls. CBD exposure, on the other hand, reduced GFAP expression only in SP relative to controls. Moreover, alterations in astrocyte morphology within the hippocampus were observed following 72 h of ∆9-THC exposure, showing significant changes in astrocyte branching compared to controls, whereas CBD’s effects were less pronounced. Additionally, ∆9-THC was found to induce clasmatodendrosis in astrocytes, representing an irreversible form of astrocytic degeneration.

#### 3.2.2. Animal Studies of GFAP Levels of Embryonal In Vivo ∆9-THC Exposure

Only two studies have investigated the impact of prenatal exposure to ∆9-THC on GFAP expression in astrocytes. Suarez et al. [24] demonstrated that in male rats, GFAP expression in the substantia nigra decreased across prepuberty, adolescence, and adulthood following prenatal exposure to ∆9-THC (from gestation day 5 to postnatal day 20). In contrast, GFAP expression in female rats increased during prepuberty, adolescence, and adulthood compared to male rats. However, GFAP expression was elevated only during prepuberty and adolescence in the substantia nigra pars reticulata (SNr) compared to controls. These findings were supported by a subsequent study examining the effects in the cerebellar cortex of rats, which indicated a reduction in GFAP expression across all ages following prenatal and perinatal exposure to ∆9-THC [25]. Importantly, these reductions in GFAP were more pronounced in males than females and persisted into later life stages, although they were partially reversible upon withdrawal of ∆9-THC.

#### 3.2.3. Animal Studies of GFAP, GLAST, and Nestin Levels of Postnatal In Vivo ∆9-THC Exposure

We reviewed nine studies investigating GFAP expression in astrocytes and nestin levels following exposure to ∆9-THC. These studies primarily utilized rodent models across various developmental stages. Among them, only one study examined early developmental stages, revealing increased GFAP and GLAST expression in the hippocampal CA1 subregion of postnatal mice after ∆9-THC exposure compared to control rats, which did not show astrocyte proliferation in the same region [32].

Five studies have focused on adolescence. One assessed GFAP expression related to learning and memory, revealing decreased hippocampal GFAP expression following ∆9-THC exposure compared to controls, with no significant difference observed in the prefrontal cortex [26]. In contrast, the other four studies documented increased GFAP expression in the hippocampus [21,22,29,31] and basolateral amygdala [31] following exposure to varying doses of ∆9-THC during adolescence. Among these, only one study examined nestin expression, reporting an increase in its levels following ∆9-THC exposure in the hippocampal area [22]. Additionally, one of these studies conducted a translational experiment that included human data to explore the relationship between ∆9-THC and cognitive factors such as decision-making, impulse control, and cognition. In their analysis of human data, they observed enhanced reward learning through computational modeling, mirroring findings observed in rodents following high doses of ∆9-THC [31].

Three studies investigated the effects in adult animals [27,30,33]. One study explored the impact of both ∆9-THC and tramadol in the striatum and cerebral cortex of adult rats [30]. ∆9-THC was administered in a dose-escalation manner (5 mg/kg, 10 mg/kg, and 20 mg/kg), resulting in elevated GFAP expression and gliosis in both brain regions compared to controls, with a more pronounced effect observed at the highest dose of ∆9-THC and in combination with tramadol. Another study focused on the cerebellum and reported increased GFAP expression and morphological changes in astrocytes in adult rats compared to controls [33]. In contrast, a separate study observed a decrease in GFAP expression following pretreatment with ∆9-THC (3 mg/kg, administered 30 min before methamphetamine exposure) in a model of methamphetamine-induced neurotoxicity; however, this study did not report a significant reduction in GFAP expression following post-treatment with ∆9-THC (3 mg/kg, administered in five doses) [27].

## 4. Discussion

This systematic review is the first to synthesize studies investigating the effect of ∆9-THC on astrocytic markers. Overall, the collective findings underscore ∆9-THC’s significant impact on astrocytes, including morphological changes, alteration in specific markers, and modulation of astrocyte maturation in preclinical studies. GFAP expression emerges as a primary target across various conditions, showing a tendency to increase based on findings from eight studies (one of which noted a GFAP level increase specifically in females) [24]. Additionally, five studies reported reductions in GFAP expression (with one study indicating a decrease specifically in males) [24]. Alterations in nestin expression were also noted in one preclinical investigation [22].

Upregulating GFAP expression is the gold standard for evaluating astrogliosis, a process where astrocytes undergo morphological and physiological changes, resulting in a gain or loss of functions in response to injury or disease [20]. The increase in GFAP expression upon Δ9-THC exposure suggests that Δ9-THC may induce a state of reactive astrogliosis, which leads to scar formation, disrupting neural connectivity and impairing brain function [23]. Moreover, the upregulation of Nestin results in increased proliferation of astrocytic phenotypes [22], whereas reductions in GLAST expression lead to excitotoxicity and neuronal damage [34]. These mechanisms contribute to glial scar formation and interfere with normal neural network function.

### 4.1. ∆9-THC Modulates GFAP Expression in Astrocytes during Early Development

Astrocytes are crucial for maintaining brain homeostasis, enhancing synapse formation, supporting metabolic activities, ensuring maintenance and plasticity, fostering maturation, and facilitating neurodevelopment [16,17]. Although astrocyte proliferation is mostly completed by early postnatal stages, the refinement of astrocytic processes continues throughout postnatal development, encompassing changes in morphology and protein expression [35].

These ongoing processes occur concurrently with a period of active synaptogenesis, during which astrocytes play a pivotal role [36]. These changes are mirrored in the expression of some astrocytic markers, such as nestin and GFAP. However, while GFAP serves as a significant structural component and aids in trafficking different proteins to the membrane, its expression undergoes significant reduction during postnatal stages compared to fibrous astrocytes [37].

It has been reported that ∆9-THC affects early development, mainly causing morphological alterations accompanied by a reduction in astrocytic reactivity [23]. Suarez et al. [24] described the impact of ∆9-THC on GFAP expression during the early phases of neurodevelopment in both male and female rats. Their findings revealed that GFAP exhibited increased expression in the substantia nigra (SNr and substantia nigra pars compacta (SNc)) of female rats compared to males following ∆9-THC treatment. This suggests a potential mechanism involving sex hormones that might contribute to the heightened vulnerability observed in male rats to GFAP reductions. Additionally, GFAP expression decreased in the SNr and SNc of male rats across all reported developmental stages (prepuberty, adolescence, and adulthood) when compared to controls. In contrast, female rats showed elevated GFAP expression at postnatal day (PND) 21 and PND30 in both SNr and SNc, with an additional increase observed only at PND30 in SNc compared to controls [24]. Another study demonstrated that that SNc expresses high levels of estrogen receptors on PND15 in female rats, compared to male rats [38]. These findings support the hypothesis that gonadal hormones influence astrocyte maturation through GFAP expression modulated by ∆9-THC, potentially due to sex-specific differences in estrogen receptor expression during early development. In a following study, Suarez et al. [25] confirmed the effects of ∆9-THC exposure on GFAP expression in the cerebellar cortex of rats. Their results revealed a pattern of GFAP reductions in early developmental stages (PND20), which were partially reversible upon ∆9-THC withdrawal (PND30). However, these effects did not persist into adulthood (PND70) and showed reductions in GFAP specifically in male rats.

Reductions in the expression of glutamine synthetase were reported across all developmental stages in both sexes. Significant changes were observed during the prepubertal and adolescent stages in male rats, and during adolescence in female rats. The decrease in glutamine synthetase expression following exposure to ∆9-THC during early developmental stages suggests an alteration in the glutamate–glutamine cycle, particularly in male rats. This indicates that chronic ∆9-THC exposure might have enduring effects on glutamate levels [39].

Indeed, ∆9-THC exposure during early developmental stages appears to exert distinct regionally specific effects on astrocytes [24,25]. This is demonstrated by reports of increased astrocytic reactivity in hippocampal regions [32]. The hippocampus, characterized by its high expression of CB_1_R [40], exhibits heightened sensitivity to ∆9-THC during early developmental stages, potentially influencing long-term neuronal formation and homeostasis. These studies suggest that ∆9-THC exposure during early development can modulate astrocyte maturation by altering the cytoskeletal structure through changes in GFAP expression and these alterations appear to depend on doses, as administration methods vary across the studies [24,25,32]. Interestingly, ∆9-THC appears to accelerate maturation in female rats by increasing GFAP expression, while conversely delaying maturation in male rats. These effects correlate well with long-term GFAP expression in adulthood following THC exposure during adolescence [21,22,29,31].

### 4.2. ∆9-THC Increases GFAP and Nestin Expression in Adulthood Rats after Adolescence ∆9-THC Exposure

The long-term effects of ∆9-THC exposure during adolescence on the hippocampus of adult rats not only result in the development of cognitive deficits but also lead to alterations in glutamate synapses, microglial activation, and increased astrocytic reactivity [21]. Ferland et al. [31] confirmed these alterations in the basolateral amygdala by observing increased GFAP expression after high-dose ∆9-THC administration (5 mg/kg), which also correlated with cognitive impairment in cannabis users. In contrast, Rubino et al. [26] reported cognitive impairments alongside reductions in postsynaptic proteins and astrocytic reactivity. One possible explanation for these different results could be the small sample size included in the study or the concentration of ∆9-THC dissolved in ethanol. Nevertheless, although one study reported an increase in cognition following ∆9-THC exposure (1.5 mg/kg), astrocytic reactivity was also observed [22]. This result suggests that GFAP expression in response to ∆9-THC administration varies depending on the dosage.

Moreover, several studies have reported the astrocytic reactivity in the drug abuse context [41]. This reactivity has been observed in the hippocampus following administration of ∆9-THC during adolescence in combination with 3,4-methylenedioxymethamphetamine (MDMA), particularly in male rats [21,22,29,31]. This finding suggests that the developing brain may be particularly susceptible to the combined effects of these substances, leading to enhanced astrocytic activation and potential neuroinflammatory responses. Conversely, when methamphetamine (METH) is administered at neurotoxic doses in combination with low-dose ∆9-THC pre-treatment (3 mg/kg) in the caudate–putamen of adult rats, a reduction in astrocytic activity is observed [27]. This indicates a potential neuroprotective or modulatory effect of ∆9-THC against METH-induced neurotoxicity. Such findings are intriguing as they point to the possibility that ∆9-THC could mitigate some of the harmful effects associated with METH exposure, potentially by modulating glial cell responses and reducing inflammation.

Considering the neurotoxic and neuroinflammatory properties of both MDMA and METH [42,43], the combined effects of ∆9-THC with other drugs on astrocytic activity appear to depend on several factors. These factors include regional variation within the brain, as different areas may exhibit varying levels of sensitivity to drug-induced changes. Additionally, the dosage of each substance plays a critical role, as does the age at which exposure occurs, with adolescent brains potentially being more vulnerable. Timing of exposure is another crucial factor, as the sequence and duration of drug administration can significantly influence the observed outcomes.

These findings underscore the complexity of drug interactions in the brain and highlight the need for further research to elucidate the mechanisms underlying these effects. Understanding how ∆9-THC interacts with other substances at the cellular and molecular levels could provide valuable insights into potential therapeutic strategies for mitigating the adverse effects of polydrug use.

### 4.3. ∆9-THC Increases GFAP Expression in Adulthood

Although the effects of ∆9-THC have been observed in adulthood following exposure during adolescence, few studies have examined its impact on astrocytic reactivity in adulthood. ∆9-THC affects both cognition and coordination (e.g., locomotor performance) and this suggests the involvement of the cerebellum [44]. Other alterations include heightened astrocytic reactivity, morphological alterations [33], and changes in the concentrations of GFAP in serum following exposure to high doses of ∆9-THC [30].

To date, studies have been limited in revealing the effects of ∆9-THC on cognition and behavioral aspects, and few studies have begun to explore these effects on astrocytes. No human studies have been undertaken to unveil whether cannabis affects astrocytic reactivity, given the current absence of techniques to measure GFAP expression in vivo in humans. However, GFAP levels have been correlated with levels of monoamine oxidase B (MAO-B) in post-mortem Parkinson’s brains [45], suggesting that MAO-B could be used as a potential marker of astrocytic reactivity [46]. This opens avenues for studying the effects of ∆9-THC in vivo for the first time in human brains of cannabis users. Indeed, several studies are investigating MAO-B levels as a potential marker of astrocytic reactivity using positron emission tomography technique in conditions such as traumatic brain injury, major depressive disorder, post-traumatic stress disorder, and alcohol use disorder, employing the radiotracer [^11^C]SL25.1188 [47,48,49,50], but also Alzheimer’s disease, using [^18^F]SMBT-1 [51]. This approach could also be applied to studying astrocytic reactivity in cannabis users [52].

## 5. Conclusions

∆9-THC appears to influence astrocytic maturation, particularly during early developmental stages, and exhibits sex-dependent effects. It tends to induce astrocytic reactivity across various brain regions, with notable effects observed in the hippocampus. These effects differ significantly across developmental stages and are closely tied to dosage levels.

## Figures and Tables

**Table 1:** Effects of ∆9-THC on astrocytic markers in animal studies.

Author	Animal Model	*n*	Stage of Development	Sex (m/f)	∆9-THC Administration, Period, Dosage	Another Drug Administered (Administration, Period, Dosage)	Methods	Brain Region and Other Markers	Astrocytic Marker Outcome
**In Vitro**									
**Landucci et al. (2022)** [23]	Rats (Wistar)	*	7 days of age	*	∆9-THC DE: 24 h, 1 μM and 72 h, 1 μM	Glutamate: 24 h, 10 mM CBD: 24 h, 10 μM and 72 h, 10 μM	IHC, WB	HIP: ⊥Synaptophysin, ↓PSD95	HIP: ↓GFAP
**Embryonal**									
**Suárez et al. (2000)** [24]	Rats ^†^	∆9-THC 30, control 30	GD 5-PND 21	∆9-THC 15/15; Control 15/15	∆9-THC PO: in dams, daily until PND 21, 5 mg/kg	N/A	IHC	Substantia nigra	Substantia nigra: ↓GFAP in male rats, ↑GFAP in females
**Suárez et al. (2002)** [25]	Rats ^†^	∆9-THC 30, control 30	GD 5-PND 20	∆9-THC 15/15; Control 15/15	∆9-THC PO: in dams, daily until PND 20, 5 mg/kg	N/A	IHC, WB	Cerebellum	Cerebellum: ↓GFAP
**Postnatal**									
**Rubino et al. (2009)** [26]	Rats (Sprague–Dawley)	∆9-THC 5, control 5	PND 35–45	10/-	∆9-THC IP: twice a day, PND 35–37, 2.5 mg/kg. PND 38–41, 5 mg/kg. PND 42–45, 10 mg/kg	N/A	WB, GI	HIP: βIII-tubulin, synaptophysin, ↓VAMP2, ↓PSD95	HIP: ↓GFAP
**Lopez-Rodriguez et al. (2013)** [29]	Rats (Wistar albino)	128 animals (48 animals for IHC analysis: Control-saline 6/6; ∆9-THC-saline 6/6; Control-MDMA 6/6; ∆9-THC-MDMA 6/6)	PND 28–45	24/24	∆9-THC (dronabinol) IP: twice a day, PND 28–34, 2.5 mg/kg^−1^. PND 35–40, 5 mg/kg^−1^. PND 41–45, 10 mg/kg^−1^	MDMA SC: in PND 30, every 5 days, twice a day, 10 mg/kg^−1^	IHC	Parietal cortex (SERT) ↑IBA-1, ↑SERT in males. Hilus area of Hippocampus: ↓CB_1_R in females (∆9-THC-MDMA)	HIP: ↑GFAP
**Castelli et al. (2014)** [27]	Rats (Sprague–Dawley)	109 animals	Adults	109/-	∆9-THC IP: 30 min before METH and at 0.5, 12, 24, 36 and 48 h after METH administration, 1 or 3 mg/kg	METH SC: every 2 h up to 6 h, 10 mg/kg. SR141716A IP: 15 min before each injection of ∆9-THC (in the 1 mg group), 1 mg/kg	IHC	PFC, caudate–putamen: ↓nNOS (∆9-THC-METH)	Caudate–putamen: ↓GFAP (∆9-THC-METH)
**Zamberletti et al. (2016)** [21]	Rats (Sprague–Dawley)	∆9-THC-behavior: 8; controls-behavior: 8. ∆9-THC-locomotor: 4, controls: 4. ∆9-THC WB: 8, controls 8. ∆9-THC-synaptosome: 5, controls: 5. ∆9-THC-microglia: 4, controls: 4	PND 35–45	58/-	∆9-THC IP: twice a day, PND 35–37, 2.5 mg/kg. PND 38–41, 5 mg/kg. PND 42–45, 10 mg/kg	N/A	WB	HIP: ↑Synaptophysin, ↑PSD95, ↑GluA1, ↑GluA2, ⊥GluN2A, ↑GluN2B, ⊥IBA-1, ⊥CD11b, ↑TNF-α, ↑iNOS, ⊥COX-2, ↓IL-10. PFC: only ↑COX-2, no alterations in the rest.	HIP: ↑GFAP
**Suliman et al. (2018)** [22]	Rats (Sprague–Dawley)	Acute ∆9-THC (0.75, 1.5, 3 mg/kg) 24, controls 8. Chronic ∆9-THC (0.75, 1.5, 3 mg/kg) 24, controls 8	5 weeks	64/-	∆9-THC IP, everyday for 7 days, 0.75, 1.5, and 3 mg/kg (acute). ∆9-THC IP, everyday for 21 days, 0.75, 1.5, and 3 mg/kg (chronic)	N/A	WB, ELISA	HIP: ↑DCX, ↑βIII-tubulin, ↑BDNF	HIP: ↑GFAP, ↑Nestin
**Abdel-Salam et al. (2019)** [30]	Rats (Sprague–Dawley)	∆9-THC 18, tramadol 18, ∆9-THC+tramadol 18, control 6	Adults	60/-	∆9-THC SC: daily for 6 weeks, 5, 10 and 20 mg/kg (in three different groups)	Tramadol SC: daily for 6 weeks, 5, 10 and 20 mg/kg (in three different groups). Tramadol + ∆9-THC SC: daily for 6 weeks, 10 mg/kg of tramadol and 5, 10, and 20 mg/kg of ∆9-THC (in three different groups)	ELISA	Whole brain: ↑UCH-L1, ↓S-100β	Whole brain: ↑GFAP
**Ferland et al. (2023)** [31]	Rats (Long–Evans)	∆9-THC (low and high doses) 55, controls 45	PND 28–59	100/-	∆9-THC IP: every third day for 32 days, 1.5 mg/kg. ∆9-THC IP: every third day for 32 days, 5 mg/kg. Adulthood: Re-exposed to acute dose of ∆9-THC IP: 0.5, 1, and 2 mg/kg	N/A	FISH	Basolateral amygdala	Basolateral amygdala: ↑GFAP mRNA
**Krassnitzer et al. (2023)** [32]	Mice ^†^	-	PND 9–16	-	∆9-THC (dronabinol) IP: 6 days, 5 mg/kg	N/A	IHC, WB, ISH	HIP (stratum radiatum): ↑S-100β	HIP: (stratum radiatum and stratum lacunosum moleculare): ↑GFAP HIP (stratum radiatum): ↑GLAST
**Mohammadpanah et al. (2023)** [33]	Rats (Wistar)	Total *n* = 20: ∆9-THC 10, control 10	Adults	20/-	∆9-THC IP: 5 days, 10 mg/kg	N/A	IHC	Cerebellum: gene expression of ⊥IL-6, ⊥HMGB1, ↑PPKAA2, ↓mTOR, ↓BECN1, ⊥ATG5, ↓LAMP2	Cerebellum: ↑GFAP

Abbreviations: ∆9-THC, delta-9-tetrahydrocannabinol; ATG5, autophagy-related protein 5; BDNF, brain-derived neurotrophic factor; BECN1, beclin 1; CB_1_R, cannabinoid receptor 1; CBD, cannabidiol; CD11b, cluster of differentiation 11b; COX-2, cyclooxygenase-2; DCX, doublecortin; DE, direct exposure; ELISA, enzyme-linked immunosorbent assay; FISH, fluorescent in situ hybridization; GD, gestation day; GFAP, glial fibrillary acidic protein; GI, Golgi Impregnation; GLAST, glutamate–aspartate transporter; GluA1 and GluA2, subunits of the α-amino-3-hydroxy-5-methyl-4-isoxazolepropionic acid receptor (AMPAr); GluN2A and GluN2B, subunits of the *N*-methyl-d-aspartate receptor (NMDAr); HIP, hippocampus; HMGB1, high mobility group box 1; IBA-1, ionized calcium binding adaptor molecule 1; IHC, immunohistochemistry; IL-10, Interleukin 10; IL-6, Interleukin 6; iNOS, inducible nitric oxide synthase; ISH, in situ hybridization; IP, intraperitoneal; IS, immunostaining; LAMP2, lysosome-associated membrane protein 2; MDMA, 3,4-methylenedioxymethamphetamine; METH, methamphetamine; m/f, male/female; mTOR, the mammalian target of rapamycin; N/A, not applicable; NMDA, N-methyl-D-aspartate; nNOS, neuronal nitric oxide synthase; PFC, prefrontal cortex; PND, postnatal day; PPKAA2, protein kinase AMP-activated subunit alpha 2; PO, per oral; PSD95, post synaptic density protein 95; S-100β, S100 calcium-binding protein β; SC, subcutaneous; SERT, serotonin transporter; SR141716A, selective cannabinoid CB1 receptor antagonist/inverse agonist; TNF-α, tumor necrosis factor α UCH-L1, ubiquitin C-terminal hydrolase L1; VAMP2, vesicle associated membrane protein 2; WB, Western blot. ⊥: no changes; ↑: increased; ↓: decreased; *: unknown or not mentioned; †: strain not specified.

## Data Availability

The data presented in this study are available on request from the corresponding author.
